# Modified DNA polymerases for PCR troubleshooting

**DOI:** 10.1007/s13353-016-0371-4

**Published:** 2016-10-28

**Authors:** Marta Śpibida, Beata Krawczyk, Marcin Olszewski, Józef Kur

**Affiliations:** Department of Molecular Biotechnology and Microbiology, Gdańsk University of Technology, ul. G. Narutowicza 11/12, 80-233 Gdańsk, Poland

**Keywords:** PCR, Native DNA polymerase, Fusion DNA polymerases, Chimeric DNA polymerases, Mutagenesis

## Abstract

PCR has become an essential tool in biological science. However, researchers often encounter problems with difficult targets, inhibitors accompanying the samples, or PCR trouble related to DNA polymerase. Therefore, PCR optimization is necessary to obtain better results. One solution is using modified DNA polymerases with desirable properties for the experiments. In this article, PCR troubleshooting, depending on the DNA polymerase used, is shown. In addition, the reasons that might justify the need for modification of DNA polymerases, type of modifications, and links between modified DNA polymerases and PCR efficiency are described.

## Introduction

DNA polymerase plays a crucial role not only in the DNA replication and repair in vivo, but also in techniques used in molecular biology, especially in polymerase chain reaction (PCR). The PCR constitutes a rapid, specific, and sensitive method for the amplification of nucleic acid sequences, and it is heavily used in research and clinical laboratories (Passarge 2007). Thermostable DNA polymerases have a broad range of applications in molecular biology, genetic engineering, and molecular diagnostics, but their usefulness depends on various features such as the error rate (fidelity), 5′ → 3′/3′ → 5′ exonuclease activity, the extension rate (kbp/min), processivity, and extra nucleotide overhang (Hubscher et al. 2010).

**Fig. 1:**
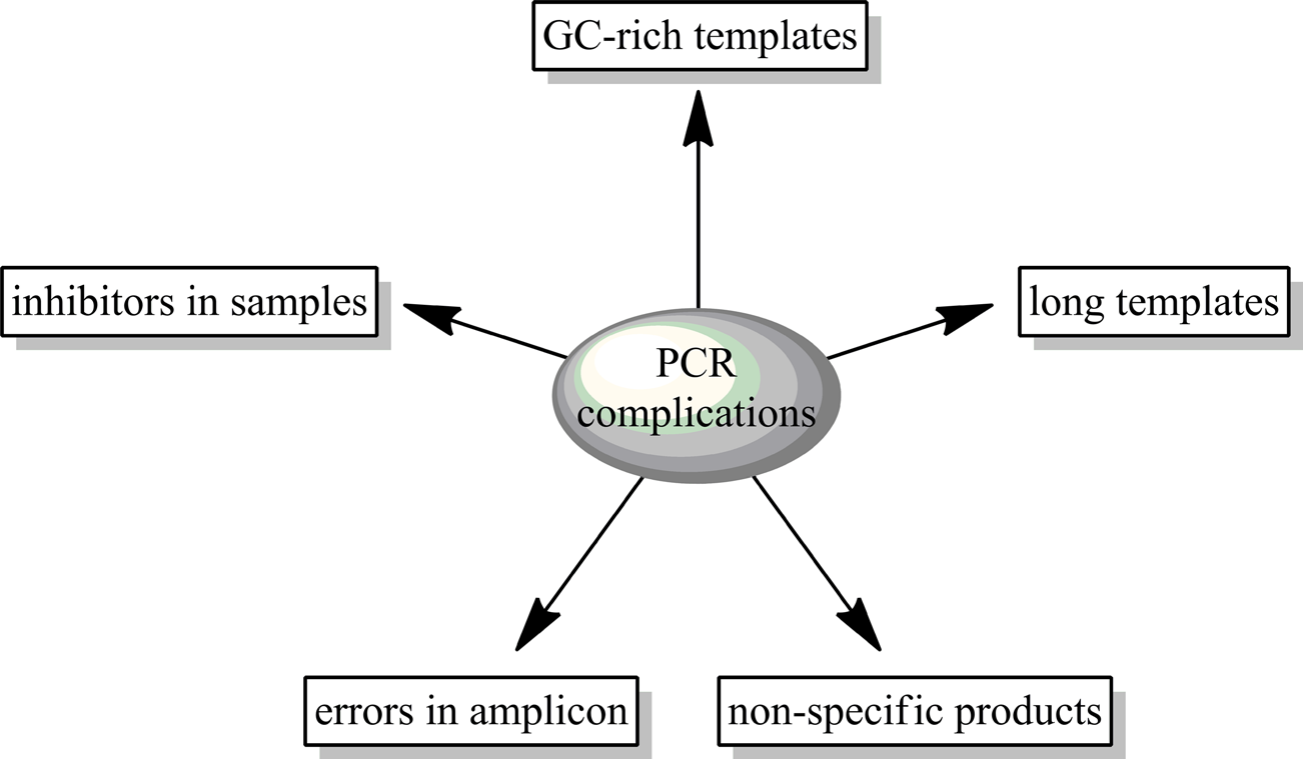
Scheme depicting the problems during PCR amplification

DNA polymerases with different features are used for problems arising during DNA synthesis. As an example of asituation when such a problem occurs, it could be amplification of DNA from environmental, clinical (e.g. blood samples), ancient, and forensic samples. The presence of inhibitors in the analyzed material cause a lack of PCR product/s or reduce the efficiency of amplification. A similar situation may occur in the case of amplification of long, GC-rich DNA fragments and looped sequences. Commercially available native polymerases are not always able to deal with all the problems in PCR. All of these impediments are described below and shown in Fig. 1.

### Inhibitors

Depending on the origin of the sample, inhibitors may be located directly in the test material (e.g. DNA isolation from blood), or as a residue from the processes used for the isolation and purification of DNA. The PCR reaction inhibitors include various kinds of organic and inorganic compounds that can interact with the nucleic acid template or block DNA polymerase directly (by polymerase degradation or by blocking the active center of polymerase) or indirectly (by blocking the active center for cofactors such as a magnesium ions) (Al-soud and Rådström 2001; Watson and Blackwell 2000).

### Difficult targets

Another problem is the amplification of long, GC-rich sequences that belong to difficult targets in PCR. The presence of refractory GC pairs makes gene amplification difficult. The presence of GC pairs above 80 % or the length of amplified gene above 10 kb is treated as difficult matrixes (Mamedov et al. 2008).

### Non-specific products

Sometimes we may have to face a different problem, such as the nonspecific amplicons in PCR. Some polymerases can show low activity at room temperature or at 4 °C. During the mixing of different reaction components, the primers may anneal nonspecifically and the enzyme may elongate these primers, resulting in a series of non-specific products. In order to prevent these non-specific products, we can use hot-start polymerase. There are techniques to create hot-start enzymes, such as manual or physical separation and antibodies, or chemical modification. In the manual technique, one of the components of reaction mixture, e.g. Mg^2+^ is added to the tube after the temperature exceeds 70 °C. Physical separation uses a barrier, e.g. a wax plug that divides reaction components. The components are mixed after the temperature is above 75 °C and the wax melts. In another method, polymerases can be inactivated by heat-sensitive antibodies or heat-labile blocking groups that are added to some amino acids. At higher temperatures, blocking groups are removed and the enzyme is activated (Primrose and Twyman 2006).

### Sequence errors

Errors in amplicons are connected with the low fidelity of polymerase. Corrective systems related to the presence of 3′ → 5′ exonuclease domain which increases the fidelity of DNA replication, which decrease the frequency of wrongly paired bases, are responsible for such errors. This problem mainly causes concerns during the amplification of products for cloning (Hubscher et al. 2010).

**Fig. 2:**
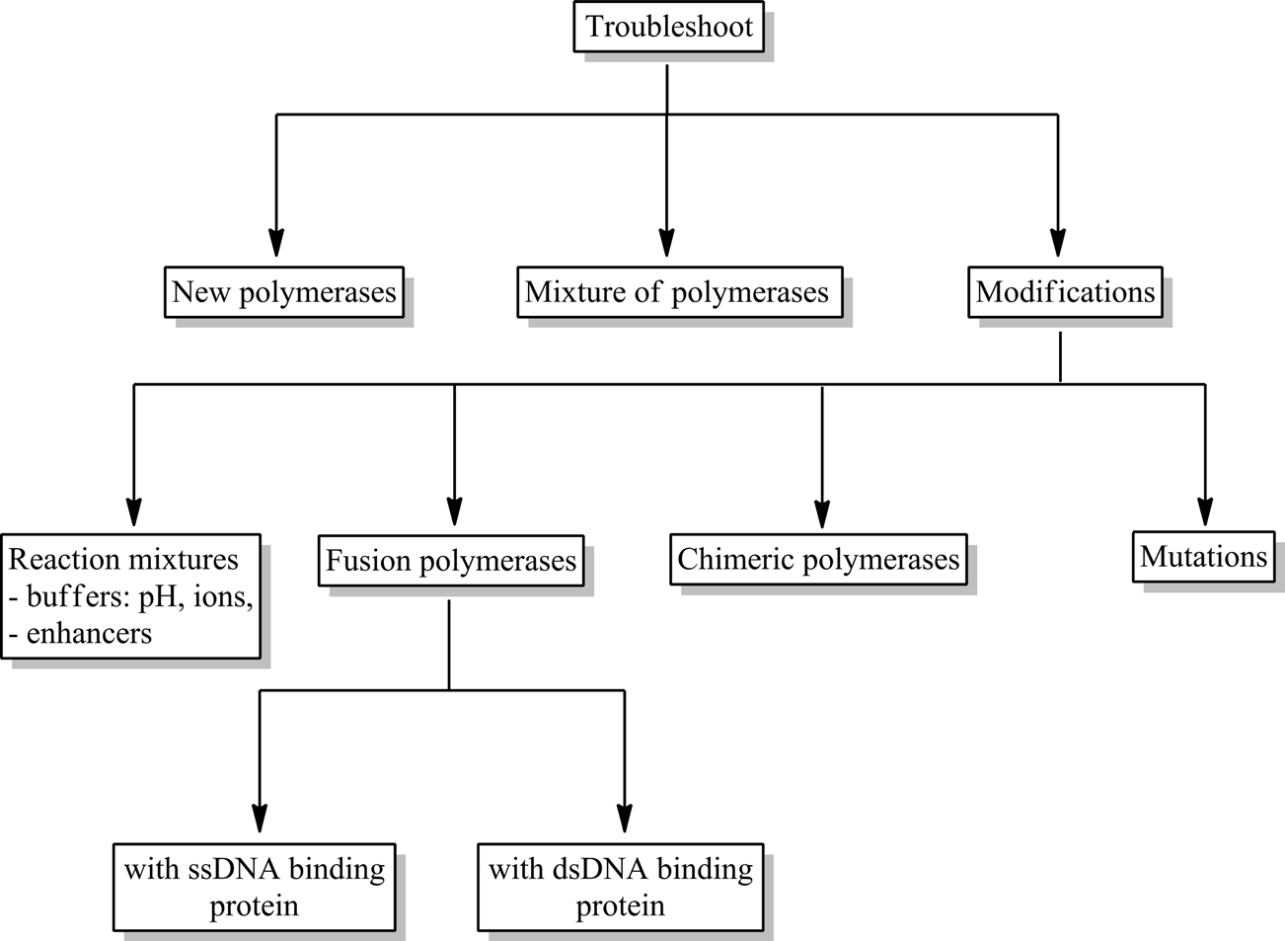
PCR troubleshooting related to DNA polymerase

All these factors mentioned above may lead to false negative results or an incorrect quantitative evaluation, as well as significantly reduce the sensitivity of the reaction. Unfortunately, commercially available polymerases are often not able to cope with all the problems in the PCR. We are still looking for new DNA polymerases of natural origin or genetically modified ones to get better parameters of the activities. In this review, we analyze the ways that can help us solve these problems. The proposed solutions are presented in Fig. 2.

## Native thermostabile DNA polymerases, their features and problems

There are two main types of thermostable DNA polymerases used in the DNA amplification technique: the A-type from bacteria belongs to, among others, the genus *Thermus* and *Thermotoga*, and the B-type from Domain Archaea and Order Thermococcales (phylum: Euryarchaeota).

A-type polymerases from the genus *Thermus* are the most popular, e.g. *Taq* DNA polymerase from *Thermus aquaticus, Tfl* from *Thermus flavus*, and *Tth* from *Thermus thermophilus*. This type has become one of the most important in commercialized molecular biology. Other A-type polymerases were isolated from bacteria *Thermotoga*, e.g. *Tma* polymerase from *Thermotoga maritima* and *Tne* from *Thermotoga neapolitana*.

The KOD polymerase from *Thermococcus kodakarensis*, *Tli* from *Thermococcus litoralis, Pfu* from *Pyrococcus furiosus, Pwo* from *Pyrococcus woesei*, and *Tfu* from *Thermococcus fumicolans* belong to B-type DNA polymerases. Many of these polymerases are available as a recombinant protein produced in *E. coli*.

DNA polymerases show different properties, depending on the presence of particular domains. The main domains occurring in these enzymes are the polymerisation domain and 3′ → 5′ or 5′ → 3′/3′ → 5′ exonuclease domain. Apart from the participation in DNA synthesis, 3′ → 5′ exonuclease domain helps the polymerase to cut out wrongly built mononucleotides from the end of newly synthesized 5′-OH DNA strand. Nuclease activity increases the fidelity of DNA replication and decreases the frequency of wrongly paired bases. However, it should be remembered that the activity of 3′ → 5′ exonuclease decreases the rate of DNA synthesis and, as a result, reduces the final efficiency of PCR. Therefore, DNA polymerases with correction activity and characterized by high accuracy are not good for amplification of long fragments of DNA. The proofreading 3′ → 5′ exonuclease activity has a second “face”; by degrading free primers at the 3′-end it causes a negative effect for the PCR (e.g. *Pfu* and *Pwo* polymerases). For this reason, hot-start should be applied to reduce the problem of primer degradation. Commercial polymerases are often produced without this activity (e.g. *Pfu* polymerase). The presence of domain 5′ → 3′ exonuclease makes it possible to cut the last bond at the end of the 5′ or a bond, a few bases away from the end of 5′. It allows for cutting out of pyrimidine dimers (Hubscher et al. 2010).

The *Taq* DNA polymerase is an example of an enzyme in which deletion of the exonuclease domain leads to the procurement of functional protein with some changed features, compared to the wild enzyme. The *Taq* polymerase without the 5′ → 3′ exonuclease domain is more thermostable, needs higher concentrations of Mg^2+^ ions for optimal activity, and is less processive than the full-length *Taq* DNA polymerase. The polymerases without the 3′ → 5′ proofreading exonuclease domain exhibit higher level of errors than the polymerases with such domain (Vainshtein et al. 1996).

Moreover there are some polymerases that provide products with blunt ends (e.g. *Pfu* polymerase) after PCR reaction or add a few additional nucleotides to the end (*Taq* polymerase). Such diverse features of DNA polymerases make it possible to use them for different purposes.

## Modifications lead to improve usefulness

To meet the demands posed by modern diagnostics, molecular biology, or genetic engineering, it is necessary to search new polymerases or improve known DNA polymerases to obtain new or better features useful in these fields. Looking for new polymerases is time-consuming. Apart from choosing an appropriate host, which is often difficult to culture, optimizing buffer conditions that polymerase may operate in is important. Modifying optimized polymerases is found to be more comfortable and quicker.

So far, the modifications implemented in the majority are based on the improved reaction buffers, PCR enhancers, and mutagenesis of the proteins. Mutations lead to the obtaining of enzymes with higher thermostability and resistance to inhibitors from clinical and environmental samples. Increasingly popular are the modifications of already-known DNA polymerases, by creating a fusion of these enzymes with proteins that may increase their processivity or fidelity.

## Improved reaction buffer and PCR enhancer

An alternative to improve the resistance of polymerases to the inhibitors may be the use of additives, enhancers, or improved buffers in PCR mix reaction. Elements added to mixtures may affect PCR reactions by increasing their sensitivity, efficiency, and specificity of the reaction, and may also reduce the inhibition for clinical or environmental samples. A distinct determination of the mechanism of the enhancers is not simple. It is assumed that it may be a sum of effects that occur in each reaction cycle, such as the impact on matrix denaturation, the hybridization of primers, or the activity of applied polymerase. The most frequent enhancers of PCR reaction are shown in Table 1.

**Table 1:** The most popular additives/enhancers of PCR reaction

PCR enhancers	Function
DMSO — (dimethylsulfoxide) (2–10 %)	Reduces intermolecular effects; during hybridization of non-stranded DNA, secondary structure is formed more loosely and access of the polymerase to structures (normally tight compress and unavailable, especially in case of areas that are rich with GC or closely related with inhibitor) is definitely easier (Musso et al. 2006)
Betaine (0.5–2 M)	Organic compound of hermaphroditic ion; effect similar to DMSO; inhibits formation of secondary structure by primers; stabilizes formation of DNA polymerase complex (Zhang et al. 2010)
DTT — (dithiothreitol) (5–40 mM)	An antioxidant; has reducing properties; has positive effect on stability of applied polymerases in PCR reaction (Nagai et al. 1998)
Albumin (100 ng/50 □l)	Blocks proteins present in a mixture, which aren’t related to the polymerase; used in PCR reactions on clinical or environmental samples and lysates; useful in case of difficult-fusible matrixes because hold stranded DNA in a denatured form (Forbes and Hicks 1996).
Formamide (max 2.5 %)	Used for DNA denaturation improvement, applied in low concentrations, inhibits formation of secondary structure (Kovárová and Dráber 2000)
Non-ionic detergents (0.1–1 %)	Triton, NP40, Tween — stabilise polymerase and DNA matrix; reduce susceptibility to inhibition by organic reagents present in the sample (Weyant et al. 1990)
Ethylene glycol (0.5–2 M)	Effect similar to betaine, experiments indicate its higher effectiveness (Zhang et al. 2009)
SSB proteins	Single-stranded DNA-binding proteins, have positive effect by reduction of dimmers of primer formation, increase amplification effectiveness and productivities (Dabrowski and Kur 1999; Dabrowski et al. 2002; Olszewski et al. 2005, 2008; Nowak et al. 2014)

## Mutagenesis

In order to provide the enzymes with new features that are not found in their native counterparts, they are subjected to mutations, the point changes in a range of gene sequence, encoding the given protein. Scientists use such possibilities of genome change and introduce mutations by a spontaneous or directed mutagenesis process.

In order to provide enzymes with new features not found in the native counterparts, they are subjected to mutations, point changes in a range of gene sequence, encoding the given protein. Scientists use such possibilities of genome change and introduce mutations in a spontaneous or directed mutagenesis process.

Adventitious mutagenesis consists of exposition of the given microorganism to the effect of physical or chemical mutagens, i.e., UV radiation, various types of ionizing radiation, alkylating agents, etc. Random mutations can also be introduced with PCR techniques. Amongst such methods, it is possible to distinguish the three most popular ones. First of them is DNA shuffling, which involves using DNase enzyme for cutting the DNA that encodes the given protein, which is supposed to be subjected to modification of random fragments of 50 to 100 bp, and then to carry out PCR reaction without using primers. DNA fragments obtained as a result of cutting, combined on the basis of complementarity principles and DNA polymerase, fill gaps between them. Mutations arise as a result of such a conducted reaction. After a few cycles of amplification, a modified gene occurs that is subjected again to a PCR reaction, this time with primers defining the end of the gene of the given protein, and enables one to clone it to the expressive vector (Stemmer 1994). Another technique for obtaining random mutation is the use of mutator strains. This method consists of cloning the gene, which is supposed to be subjected to mutagenesis into plasmid. Then, a transformation into modified *E. coli* strain is carried out, which was deprived of one or few basic repair trails of DNA (*mutS*, *mutD*, or *mutT*). The lack of a DNA repair system results in changes during replication in the genetic material entered into the strain; as a result, the target gene undergoes random mutations. A disadvantage of this method is that the modified *E. coli* strain quickly loses viability, because the genes encoding basic vital functions also undergo mutations that are devoid of one of repair trails, which leads to random changes during gene replication. It is also possible to use the “error-prone PCR” technique. It consists of applying DNA polymerase with reduced replication fidelity and placing it in the reaction buffer, which is modulated in such a way that the response was non-specific. The enzyme, as a result of small catalysis specificity, introduces mutations into new stranded DNA. The control of the composition in the reaction mixture allows for adjustment of error frequencies entered into newly synthesized DNA strands. The frequency of mutation led by DNA polymerase is about 1–3 changes on 1 kbp (Hanson-Manful and Patrick 2013).

Targeted mutations primarily use PCR reactions; thus, changes in the sequence can be led in a controlled way. In such techniques, modified primers are applicable, both external and internal, which have non-complementary nucleotides to the matrix, in this way introducing the desired mutation (Reikofski and Tao 1992; Ho et al. 1989).

Techniques of directed and adventitious mutagenesis have been used in the creation of polymerases with improved useful features in molecular diagnostics and genetic engineering. The most common polymerase subjected to such modifications is the best-known and most applied *Taq* polymerase from *Thermus aquaticus*. Mutations leading to changes in replication fidelity of DNA by polymerase include the highly preserved O-helix area. This is an area consisting of 12 amino acid residues from 659 to 671 amino acids, which is also a part of the space that binds the enzyme with DNA and nucleotides built to a new strand. Research shows that changes in four of 12 amino acid residues (Arg-659, Lys-663, Phe-667 and Tyr-671) lead to significant deterioration in replication fidelity by *Taq* DNA polymerase. Additionally, a change of 667 Phe on Tyr causes more frequent activation of ddNTP to newly-synthesized stranded DNA, which is desired in techniques of Sanger’s sequencing method (Suzuki et al. 1997, 2000). Obtaining enzymes with reduced replication fidelity is also applied in mutagenesis techniques with error-prone PCR, where such polymerases are desired.

Mutations introduced in amino acidic residues Glu-742 and Ala-743 in the subdomain of fingers of *Taq* DNA polymerase turn out to be very important in the affinity of the polymerase to the DNA matrix. Studies suggest that this is critical for elongation ability of the enzyme. Examined variants of mutations each time have led to an increase in the affinity of DNA and faster extending primers in comparison to a wild strain. Mutations in this range constitute a potential to obtain polymerases with improved processivity, productivities, and reaction rate (Yamagami et al. 2014).

In view of the increasing need to conduct PCR reactions, where in the reactionary mixture there are many compounds that are polymerase inhibitors, research has been conducted to find new (mutated) polymerases, which will not be sensitive to their presence. The first positive results, which resulted in the implementation of a new product, concerned modification of *Taq* DNA polymerase. A lot of mutants were tested and among those selected were the ones with the highest application potential. They were characterized with a much higher resistance to inhibitors included in blood with regard to wild proteins, and enabled efficient receiving amplicons at 20 % blood concentration in the reactionary mixture. Mutations were carried out in the area of zinc fingers in positions 708 and 707. Mutations in position 708 ensured resistance to the blood sample. Glutamic acid found in this position in the native polymerase was exchanged into valine, lysine, leucine, and tryptophan (Kermekchiev et al. 2009).

Changes of amino acid residues in 706, 707, and 708 may lead to formation of hot-starting polymerases. An exchange of tryptophan in position 706 to arginine, isoleucine 707 to leucine, and glutamic acid 708 to aspartic, leads to a decrease in the activity of protein at low temperatures, which increases the specificity of the PCR reaction (Kermekchiev et al. 2009).

## Chimeric DNA polymerases

**Fig. 3:**
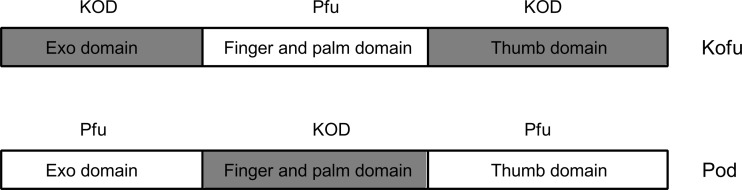
Domains layout in chimeric polymerase *Kofu* and *Pod*. KOD- domain derived from *Thermococcus kodakarensis* polymerase; *Pfu* — domain derived from *Pyrococcus furiosus* polymerase

Chimeric DNA polymerases are proteins whose amino acid sequence consists of subsequences from at least two separate proteins. A covalent combination of these subsequences by using genetic engineering techniques causes an expression of the functional protein. Chimeric bacterial DNA polymerase is an example of such modification, which was formed as a result of fusion amino acidic subsequences from *Tth* and *Taq* DNA polymerases. On the N-end of the amino acidic chain in this enzyme there is a fragment of *Tth* DNA polymerase, but the C-end is built from fragments of *Taq* DNA polymerase (Ignatov et al. 2009). Considering the unique characteristics of *Tth* DNA polymerase and *Taq* DNA polymerases, it was decided to single out the part with the most desirable properties and to connect it to obtain a functional, chimeric protein. An amino acidic sequence of chimeric thermostable *Tth*–*Taq* DNA polymerases consists of the part of N-end from *Tth* polymerase (4–600 amino acidic residues) and C-end from *Taq* DNA polymerase (556–834 amino acidic residues) (Ignatov et al. 2009). The obtained chimeric *Tth*–*Taq* DNA polymerase is characterized with desirable characteristics of both *Tth* DNA polymerase and *Taq* DNA polymerase. It shows high replication effectiveness of long DNA sequences. Productivity of chimeric enzyme is at least 5 times higher than for *Taq* DNA polymerase, and comparable with *Tth* DNA polymerase. Chimerical enzyme is at least 6 times more sensitive to the presence of incongruity on 3′-end of primer than *Tth* polymerase, and comparatively sensitive to Taq DNA polymerase. The specificity of amplification reaction is much greater than using *Tth* DNA polymerase, and comparable with *Taq* DNA polymerase (Ignatov et al. 2009). Another example of chimeric DNA polymerases are *Kofu* and *Pod* enzymes. They were obtained by the exchange of domains between KOD DNA polymerases (*Thermococcus kodakarensis*) and *Pfu* DNA polymerases (*Pyrococcus furiosus*). For *Kofu* DNA polymerase, N-end domain of exonuclease and C-end are derived from KOD polymerase, but the domain of metacarpus and fingers were from *Pfu* DNA polymerase (Fig. 3). The Kofu chimeric DNA polymerase is characterized with a similar rate of extending primers to KOD DNA polymerase (106–138 nt/s), processivity (∼300 nt/s), and thermal stability, while the replication fidelity is similar to *Pfu* DNA polymerase (2.0 × 10^−6^). The *Pod* DNA polymerase is by analogy a connection with *Pfu* DNA polymerases and KOD; in addition, it is characterized with a rate of extending primers (25 nt/s), processivity (>20 nt), and similar thermal stability to *Pfu* polymerase, while replication fidelity is similar to KOD DNA polymerase (4.45 × 10^−6^) (Faurholm et al. 2012).

The next example of chimeric DNA polymerase is an enzyme which was formed as a result of domain exchange between *Taq* DNA polymerase and *Tma* DNA polymerase from *Thermococcus marinus*. Apart from the fact that this is a chimeric protein, it also has four mutations that affect its functions. The N-end of this enzyme is built from amino acidic residues of 1 to 190 from *Taq* DNA polymerase. Within this area, there is a 5′ → 3′ exonuclease domain. Its activity is 1,000-fold reduced by the G46D mutation. The C-end area consists of amino acidic residues of 191 to 893 from *Tma* polymerase. In this area, among others, a 3′ → 5′ exonuclease domain is present, which is inactivated by introducing point mutations of D323A and E325A. In addition to all modifications of DNA polymerase mentioned above, it also has a mutation of F730Y, which causes this enzyme to activate ddNTP more willingly to newly synthesized stranded DNA (Faurholm et al. 2012). Such an enzyme is a useful tool in DNA sequencing techniques with Sanger’s method. Such a chimeric DNA polymerase is characterized with the improved ability to activate ddNTP in newly synthesized stranded DNA (Hamilton et al. 2001). Moreover, it increases reproducibility of DNA sequencing peak heights, in particular, with a ddNTP dye marker in the sequencing reaction cycle. It reduces the pyrophosphorolysis rate of marked with ddNTP dyes, and improves introduction of dITP (Gelfand and Reichert 2001).

## Fusion DNA polymerases

One of the most important steps in polymerization activity of DNA polymerase, which is responsible for their final efficiency, is the initiation step connected with the binding to the matrix DNA. Therefore, it is reasonable to modify well-known DNA polymerases to facilitate their binding to a polymerized DNA strand. An example of such modification may be the creation of fusion of DNA polymerases with proteins, which naturally bind to single- or double-stranded DNA. However, only a few examples have been described in scientific literature (Hubscher et al. 2010).

### Fusion with a double-stranded DNA binding protein

**Fig. 4:**
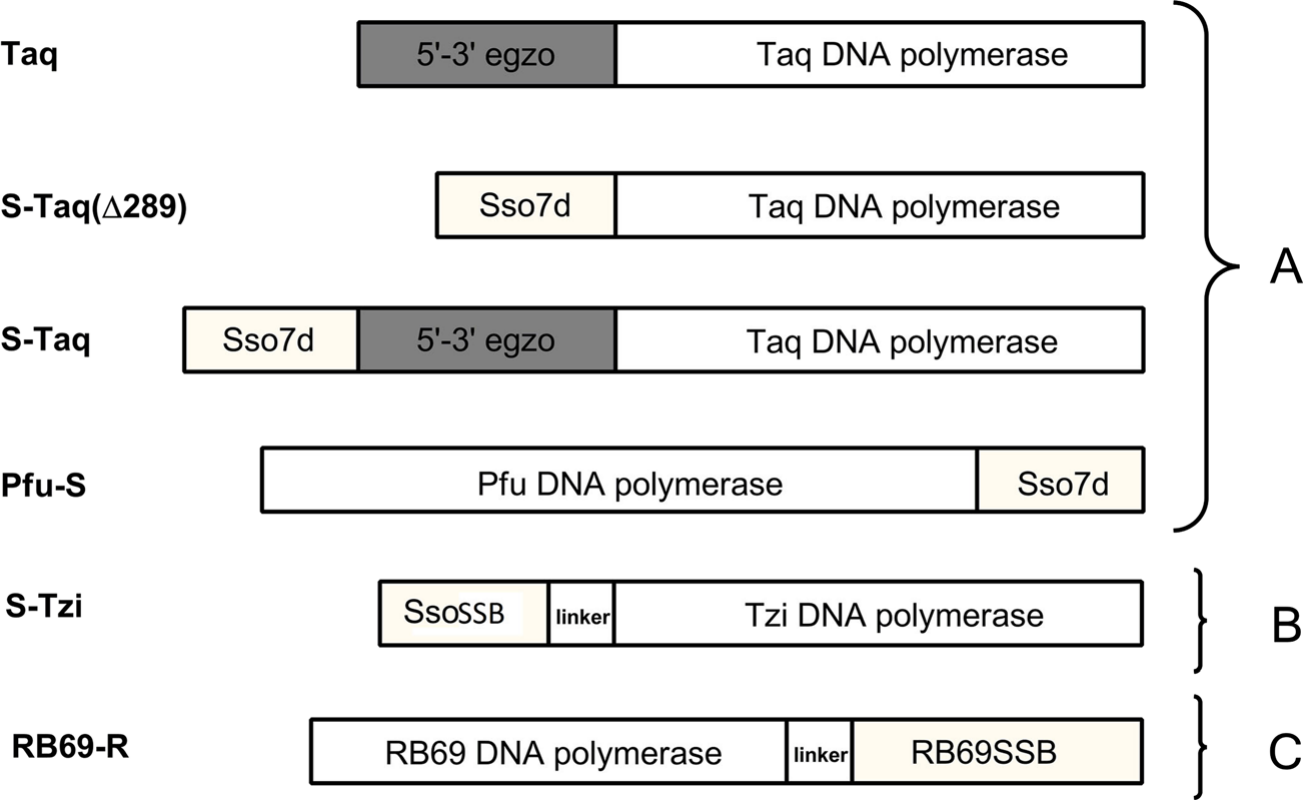
Schematic diagram of the fusion DNA polymerases: *Taq* and *Pfu* with Sso7d protein (**a**), *Tzi* with *Sso*SSB protein (**b**), *RB69* with RB69SSB protein (**c**)

The only fusion of *Taq* DNA polymerase described in literature is fusion with Sso7d protein (Wang et al. 2004). This is a protein from hyperthermophilic archaebacteria *Sulfolobus solfataricus* that binds double-stranded DNA. It has a small mass, about 7 kDa; in solution it is found as the monomer. Its function in the native microorganism is the stabilization of the genomic DNA and thereby facilitation of the action of DNA polymerase (Gao et al. 1998). Wang et al. (2004) conducted fusion of this protein with *Taq* DNA polymerase, *TaqStoffel* and *Pfu* DNA polymerase, and then they compared processivity of modified polymerases to native polymerase. For fusion with *Taq* DNA polymerase and *TaqStoffel*, the Sso7d protein was placed on the N-end of polymerases and combined directly with the enzyme. However, in *Pfu* DNA polymerase the Sso7d protein was placed on the C-end via 3-amino-acids linker (Gly–Thr–His) . Variants of fusion polymerases are presented in Fig. 4a. Studies showed that native *Taq* DNA polymerase was characterized with greater processivity than polymerase with the deletion of 5′ → 3′ exonuclease domain. Introduction of Sso7d protein in a place of “exo” domain led to an increase in processivity, as compared to polymerase without fusion. However, native *Taq* DNA polymerase with additional fusion protein still exceeds processivity of fusion polymerase with deletion. For *Pfu* polymerase, there is a preserved tendency in processivity improvement of fusion proteins compared to the native ones. A general increase in processivity in all conducted fusions was up to 17 times in relation to the appropriate native polymerases. Moreover, *Pfu* DNA polymerase from archaeon is the representative of a completely different family of polymerases than the bacterial *Taq* DNA polymerase. The evidence that for both fusions an increase in processivity is observed, may suggest the universality of applied modification regardless of the type of polymerases used (Wang et al. 2004). Further studies with a fusion of *Tpa* DNA polymerase from *Thermococcus pacificus* bacterium with the same Sso7d protein confirmed this fact, where a similar significant increase in processivity and productivities of polymerase was noticed, without the negative effect on catalytic action or change of the enzyme stability (Lee et al. 2010). With the Sso7d protein, there was also a combined KOD polymerase from hyperthermophilic Archaea, *Thermococcus kodakaraensis*, KOD1. Fusion polymerase leads to an increase in productivities and processivity in relation to the native KOD polymerase, without a significant effect on the thermal stability of the obtained polymerase (Wang et al. 2015).

### Fusion with a single-stranded DNA binding protein

In the European patent from 2013 (Lee et al. 2013), DNA polymerase from *Thermococcus zilligi* in fusion with SSB protein from *Sulfolobus solfataricus* (Haseltine and Kowalczykowski 2002) was described (Fig. 4b). The SSB protein is located on N-end polymerases, and the used linker partly differs by the amino acids sequence: Gly–Ser–Gly–Gly–Val–Asp. The study conducted on the modified polymerase indicates a significant increase in its productivities, fidelity, and processivity in relation to the native *Tzi* DNA polymerase (Lee et al. 2013).

In the literature, it is also possible to find an example of fusion DNA polymerase with single-stranded DNA-binding protein. Improvement of enzyme features is described in the example of DNA polymerase of RB69 bacteriophage in combination with its native SSB protein. The RB69SSB is a small, monomeric protein, which has a great significance in the proper action of DNA polymerase of this bacteriophage. In the examined fusion, the protein was placed on the C-end of polymerase via 6-amino-acids linker: Gly–Thr–Gly–Ser–Gly–Thr (Fig. 4c) (Sun et al. 2006).

The presence of the linker, contrary to stiff direct connection, provides fusion protein with certain flexibility and relatively free arrangement in relation to the polymerase. This helps to avoid a possible steric hindrance, which may be significant in binding to DNA and its polymerization. As a result of the conducted fusion, the obtained polymerase shows much improved features used in the diagnostics or molecular biology. New polymerase shows a 6-fold increase of affinity to the DNA matrix and a 7-fold improvement in processivity while maintaining the fidelity. Moreover, its ability to the amplification of long DNA fragments has increased (Sun et al. 2006).

As for now, commercially there is only one available fusion DNA polymerase, *Pfu* with Sso7d protein (Table 2). Research that has been carried out so far indicates that the connection of small protein to a polymerase can significantly improve the functional properties without affecting its stability or activity.

**Table 2:** The list and characteristic of commercially available fusion polymerases

Trade name	Structure	Effect
Phusion High-Fidelity (Thermo Scientific)	*Sso7d + Pfu*	Increased fidelity and processivity, amplification of longer DNA fragments
Hercules II Fusion (Agilent Technologies)	*Sso7d + Pfu*	Amplification of matrixes that are rich in GC, high sensitivity, increased processivity
Phusion (NEB)	*Sso7d + Pfu*	Greater fidelity, rate, and specificity, amplification of matrixes that are rich in GC
iProof ™ High-Fidelity DNA Polymerase (Bio-Rad)	*Sso7d + Pfu*	Amplification of longer DNA fragments, DNA processivity and fidelity

## Application of native and modified DNA polymerases

The polymerases in bacteria, archaea, and eukaryotes have a similar structure. On the other hand, DNA polymerases have a variation in the rate of catalysis, processivity, the presence or absence of interacting protein subunits, or demonstrating nucleolytic activity. The specific application often determines the choice of specific polymerase.

Presence of proofreading domain is important when PCR products are used to prepare cloning inserts or for the detection of mutations. For such applications, *Pwo* or Phusion DNA polymerases are recommended. Error rates for these polymerases are at least 10 times lower than the error rate observed with *Taq* polymerases (McInerney et al. 2014).

Amplification of longer DNA fragments is possible for the *Tfu* polymerase from *Thermococcus fumicolans* (products up to 10 kb). The KOD polymerase can also be used to amplify long products because it has a 10–15-fold higher processivity and greater elongation capability than the *Pfu* DNA polymerase (Takagi et al. 1997).

The *Pfu* DNA polymerase from the archaebacterium is thermostable, even at 95 °C for 4 h, and is recommended for GC-rich templates. Effective polymerase for GC-rich templates is also fusion polymerase with Sso7d protein.

The thermostable Y-DNA polymerases that are used for PCR of damaged or ancient DNAs belong to a novel family of DNA polymerases (McDonald et al. 2006). The DNA polymerases from the Y-family are responsible for the synthesis of DNA strands over the side of strand damage. The structure of these polymerases is free of 3′ → 5′ exonuclease activity, and they have greater tolerance to DNA damage. Representatives of this family are DNA polymerase IV from *Sulfolobus solfataricus* and DNA polymerase IV from *E. coli* (Lehmann 2006).

A good solution is a mixture of polymerases of different properties in appropriate ratios to each other, e.g., *Taq* and *Tpe* (from *Thermococcus peptonophilus*) DNA polymerases in a 31:1 ratio that could be used for PCR amplification of targets between 6 and 8 kb and with maximum of up to 15 kb (Lee et al. 2010) or a mixture of KOD and KOD (exo-) destined for the long complex targets up to 15 kb (Nishioka et al. 2001). Similarly, a *Taq* and *Pfu* polymerases mixture in a 16:1 ratio amplified the DNA targets to more than 30 kb (Cline et al. 1996).

Some polymerases are recommended for detecting microorganisms from samples of blood, cheese, feces, soil, or meat in PCR. These samples contain large amounts of inhibitors. The research indicates that *Taq* DNA polymerase is inhibited by components from biological samples, but *Tfl* from *Thermus flavus*, *Tli* from *Thermus litoralis*, *Tth* from *Thermus thermophilus*, and *Pfu* DNA polymerases are several times more resistant. For example, *Taq* DNA polymerase is completely inhibited in the presence of 0.004 % (*v/v*) blood in the PCR, but the polymerases listed above amplified with 20 % of blood in the mixture (Al-soud and Rådström 1998).

Fusion polymerases may have a huge application, as tools in diagnostics, molecular biology, or genetic engineering. Commercially available fusion polymerases (shown in Table 2) leads to improved fidelity, processivity, and amplified GC-rich sequences. Because there are many problems during amplification of different DNA samples, biotechnological companies construct new and improved versions of DNA polymerases in response to these problems. The most popular problems, their solutions, and proposed DNA polymerases are presented in the Table 3.

**Table 3:** The most popular problems, reasons and their solutions with proposed DNA polymerases

Problem	Reason	Solution	Recommended DNA polymerase	
			native	modified
Non-specific products	activity of polymerase at RT	used Hot Start polymerase	–	*Taq* W706R I707L Z708B
Blood or environmental samples	presence of inhibitors	used inhibitor-resistant polymerases	*Tfl*, *Tli*,*Tth*,*Pfu*	*Taq* Z708V,K,L
Errors in sequence	polymerase with low fidelity	improved polymerase fidelity	*Pfu*	Phusion SsoSSB-Tzi Kofu
Long amplicons	polymerase with low processivity	improved polymerase processivity	mixture of *Taq* and *Tpe* mixture of *KOD* and *KOD* (exo-) *Thermococcus fumicolans*	Phusion RB69-RB69SSB Sso-Taq Pfu-Sso7d SsoSSB-Tzi Sso7d-KOD Tth-Taq
GC-rich templates	presence of high-melting GC pairs	high-thermostabile polymerase	*Pyrolobus fumarius*	Phusion

## Conclusion

To sum up, the introduction of PCR technology has made a significant impact in molecular research and diagnostics, since enormous amounts of data can be obtained within a short research time. Developments such as the new native polymerases or fusion and chimeric DNA polymerases have revolutionarily simplified the process of detection of DNA and RNA from difficult samples.

## References

[CR1] Al-soud WA, Rådström P (1998). Capacity of nine thermostable DNA polymerases to mediate DNA amplification in the presence of PCR-inhibiting samples. Appl Environ Microbiol.

[CR2] Al-soud WA, Rådström P (2001). Purification and characterization of PCR-inhibitory components in blood cells. J Clin Microbiol.

[CR3] Cline J, Braman JC, Hogrefe HH (1996). PCR fidelity of Pfu DNA polymerase and other thermostable DNA polymerases. Nucleic Acids Res.

[CR4] Dabrowski S, Kur J (1999). Cloning, overexpression, and purification of the recombinant his-tagged SSB protein of Escherichia Coli and use in polymerase chain reaction amplification. Protein Expr Purif.

[CR5] Dabrowski S, Olszewski M, Piatek R, Brillowska-Dabrowska A, Konopa G, Kur J (2002). Identification and characterization of single-stranded-DNA-binding proteins from Thermus thermophilus and Thermus aquaticus — new arrangement of binding domains. Microbiology.

[CR6] Faurholm B, McEwan P, Bourn W, Rush G (2012) Chimeric DNA polymerases. US Patent 20120115188 A1

[CR7] Forbes BA, Hicks KE (1996). Substances interfering with direct detection of mycobacterium tuberculosis in clinical specimens by PCR: effects of bovine serum albumin. J Clin Microbiol.

[CR8] Gao YG, Su SY, Robinson H, Padmanabhan S, Lim L, McCrary BS, Wan AH (1998). The crystal structure of the hyperthermophile chromosomal protein Sso7d bound to DNA. Nat Struct Biol.

[CR9] Gelfand DH, Reichert FL (2001) Mutant chimeric DNA polymerase. US Patent 6228628 B1

[CR10] Hamilton SC, Farchaus JW, Davis MC (2001). DNA polymerases as engines for biotechnology. BioTechniques.

[CR11] Hanson-Manful P, Patrick WM (2013). Construction and analysis of randomized protein-encoding libraries using error-prone PCR. Methods Mol Biol.

[CR12] Haseltine CA, Kowalczykowski SC (2002). A distinctive single-strand DNA-binding protein from the archaeon *Sulfolobus Solfataricus*. Mol Microbiol.

[CR13] Ho SN, Hunt HD, Horton RM, Pullen JK, Pease LR (1989). Site-directed mutagenesis by overlap extension using the polymerase chain reaction. Gene.

[CR14] Hubscher U, Spadari S, Villani G, Maga G (2010). DNA polymerases — discovery, characterization and functions in cellular DNA transactions.

[CR15] Ignatov K, Kramarov V, Billingham S (2009) Chimeric DNA polymerase. US Patent 20090209005 A1

[CR16] Kermekchiev MB, Kirilova LI, Vail EE, Barnes WM (2009). Mutants of Taq DNA polymerase resistant to PCR inhibitors allow DNA amplification from whole blood and crude soil samples. Nucleic Acids Res.

[CR17] Kovárová M, Dráber P (2000). New specificity and yield enhancer of polymerase chain reactions. Nucleic Acids Res.

[CR18] Lee JI, Cho SS, Kil EJ, Kwon ST (2010). Characterization and PCR application of a Thermostable DNA polymerase from *Thermococcus pacificus*. Enzyme Microb Technol.

[CR19] Lee JE, Potter RJ, Mandelman D (2013) SSB - polymerase fusion proteins. EP Patent 1934372 B1

[CR20] Lehmann AR (2006). New functions for Y family polymerases. Mol Cell.

[CR21] Mamedov TG, Pienaar E, Whitney SE, TerMaat JR, Carvill G, Goliath R, Subramanian A, Viljoen HJ (2008). A fundamental study of the PCR amplification of GC-rich DNA templates. Comput Biol Chem.

[CR22] McDonald JP, Hall A, Gasparutto D, Cadet J, Ballantyne J, Woodgate R (2006). Novel thermostable Y-family polymerases: applications for the PCR amplification of damaged or ancient DNAs. Nucleic Acids Res.

[CR23] McInerney P, Adams P, Hadi MZ (2014). Error rate comparison during polymerase chain reaction by DNA polymerase. Mol Biol Int.

[CR24] Musso M, Bocciardi R, Parodi S, Ravazzolo R, Ceccherini I (2006). Betaine, Dimethyl Sulfoxide, and 7-Deaza-dGTP, a powerful mixture for amplification of GC-rich DNA sequences. J Mol Diagn.

[CR25] Nagai M, Yoshida A, Sato N (1998). Additive effects of bovine serum albumin, dithiothreitol and glycerolon PCR. Biochem Mol Biol Int.

[CR26] Nishioka M, Mizuguchi H, Fujiwara S, Komatsubara S, Kitabayashi M, Uemura H, Takagi M, Imanaka T (2001). Long and accurate PCR with a mixture of KOD DNA polymerase and its exonuclease deficient mutant enzyme. J Biotechnol.

[CR27] Nowak M, Olszewski M, Śpibida M, Kur J (2014). Characterization of single-stranded DNA-binding proteins from the psychrophilic bacteria Desulfotalea psychrophila, Flavobacterium psychrophilum, Psychrobacter arcticus, Psychrobacter cryohalolentis, Psychromonas ingrahamii, Psychroflexus torquis, and Photobacterium profundum. BMC Microbiol.

[CR28] Olszewski M, Rebała K, Szczerkowska Z, Kur J (2005). Application of SSB-like protein from *Thermus aquaticus* in multiplex PCR of human Y-STR markers identification. Mol Cell Probes.

[CR29] Olszewski M, Mickiewicz M, Kur J (2008). Two highly thermostable paralogous single-stranded DNA-binding proteins from Thermoanaerobacter tengcongensis. Arch Microbiol.

[CR30] Passarge E (2007). Color atlas of genetics.

[CR31] Primrose SB, Twyman RM (2006). Principles of gene manipulation and genomics.

[CR32] Reikofski J, Tao BY (1992). Polymerase chain reaction (PCR) techniques for site-directed mutagenesis. Biotechnol Adv.

[CR33] Stemmer WP (1994). DNA shuffling by random fragmentation and reassembly: in vitro recombination for molecular evolution. Proc Natl Acad Sci U S A.

[CR34] Sun S, Geng L, Shamoo Y (2006). Structure and enzymatic properties of a chimeric bacteriophage RB69 DNA polymerase and single-stranded DNA binding protein with increased processivity. Proteins.

[CR35] Suzuki M, Avicola AK, Hood L, Loeb LA (1997). Low fidelity mutants in the O-Helix of *Thermus Aquaticus* DNA polymerase I. J Biol Chem.

[CR36] Suzuki M, Yoshida S, Adman ET, Blank A, Loeb LA (2000). *Thermus Aquaticus* DNA polymerase I mutants with altered fidelity. Interacting mutations in the O-Helix. J Biol Chem.

[CR37] Takagi M, Nishioka M, Kakihara H, Kitabayashi M, Inoue H, Kawakami B, Oka M, Imanaka T (1997). Characterization of DNA polymerase from Pyrococcus Sp. Strain KOD1 and its application to PCR. Appl Environ Microbiol.

[CR38] Vainshtein I, Atrazhev A, Eom SH, Elliott JF, Wishart DS, Malcolm BA (1996). Peptide rescue of an N-Terminal truncation of the Stoffel fragment of Taq DNA polymerase. Protein Sci.

[CR39] Wang Y, Prosen DE, Mei L, Sullivan JC, Finney M, Horn PBV (2004). A novel strategy to engineer DNA polymerases for enhanced processivity and improved performance in vitro. Nucleic Acids Res.

[CR40] Wang F, Li S, Zhao H, Bian L, Chen L, Zhang Z, Zhong X, Ma L, Yu X (2015). Expression and characterization of the RKOD DNA polymerase in Pichia Pastoris. PLoS One.

[CR41] Watson RJ, Blackwell B (2000). Purification and characterization of a common soil component which inhibits the polymerase chain reaction. Can J Microbiol.

[CR42] Weyant RS, Edmonds P, Swaminathan B (1990). Effect of ionic and nonionic detergents on the Taq polymerase. BioTechniques.

[CR43] Yamagami T, Ishino S, Kawarabayasi S, Ishino Y (2014). Mutant Taq DNA polymerases with improved elongation ability as a useful reagent for genetic engineering. Front Microbiol.

[CR44] Zhang Z, Yang X, Meng L, Liu F, Shen C, Yang W (2009). Enhanced amplification of GC-rich DNA with two organic reagents. BioTechniques.

[CR45] Zhang Z, Kermekchiev MB, Barnes WM (2010). Direct DNA amplification from crude clinical samples using a PCR enhancer cocktail and novel mutants of Taq. J Mol Diagn.

